# Everybody's Page

**Published:** 1890-02-01

**Authors:** 


### Everybody's Page.

In New York one case of infant death was reported as "sat on by father."

It is becoming a common practice in Spain, in the treatment of wines during or after fermentation, to acidify them with tartaric, nitric, citric, sulphuric, or phosphoric acid.

Small comfort to those who are still suffering the torments of influenza to know that the germ detectives have at last hunted down the villain bacillus, and have him in Vienna. Oh! that he had been caught and condemned to penal servitude long ago.

The height of waves at sea has long been a much disputed subject, but scientific testimony goes to show that storm waves are often forty and also sixty or seventy feet high; it is probably some of the latter height that have been making such havoc among the Atlantic liners recently.

One of the staff of St. Bartholomew's Hospital has called the attention of the public to the numerous accidents caused by tramcars, owing to the inability of the driver of a tramcar to alter his course, as the driver of an ordinary vehicle can do. It is really astonishing that not more accidents are caused by omnibusses from the way in which many of the drivers race their horses along the streets, quite regardless of the fact that the foot-passenger has some little right to the road as well as the mighty Jehu.

All work and no play makes Jack a dull boy, excepting John Chinaman, and one of the chief things that makes him so unpopular in the labour market is his utter nervelessness. Nothing upsets the pig-tailed man. He is quite content to go on day by day in the same monotonous round of work, where a European would most probably be getting nervous and irritable, and after a hard day's work he will roll himself up anyhow and anywhere, and sleep like the proverbial top. Happy Chinaman! Perhaps he thinks himself hardly treated in having no nerves, and so he smokes opium and tries to get some.

A lady lately wrote a very useful letter in *Work and Leisure*, which would be of great help to anybody of moderate means who was obliged to winter in the south. She mentions, amongst other things, that Nice is so much less expensive than Cannes, and with a little patience in hunting about, and a bargain with the landladies, a small furnished flat, or bedroom, sitting-room and kitchen, may be found for 60 francs a month, paid in advance. The great secret in living cheaply at Nice is to try and get on in French fashion, and not live in the English way, to market yourself, and employ a carrier woman to bring things back twice a week or so. The English in Nice, says the letter, are sociable and friendly.

The time has arrived when people who wish to look well do not attempt any more to cut their own finger nails. They humbly reflect how hideous their nails have looked in the past, and resolve to do better in the future, and so, putting away their nail scissors with which they have hitherto been content, they hie them to some famous man to execute this part of their toilet for them. H. P. Truefitt, for instance, announces that he has "American Manicure Courts" (*courts* sound a trifle chilly in January), "where he has secured the services of the most highly-skilled staff in Europe," who cleanse, and cut, and polish nails to such an extent that the owners of the nails scarcely recognise them. Some wit says that "the manicure is expert by proxy; he has his business at other people's finger ends."

"Did you never fight a duel, doctor?" "Never; what satisfaction could I derive from killing a fellow-creature?" "Oh! I see, you're so used to it."

A great number of people go to bed without remembering to clean their teeth. When we consider how much food collects in the crevices during the day we know how injurious it must be to let it remain there so many hours; nothing helps teeth to decay sooner.

"Faith healing" does not meet with very light treatment in Brooklyn. Three "faith cure fanatics" there lately refused to allow a mother and child to take some medicine, and, in consequence, they were fined two hundred, a hundred and fifty, and one hundred dollars respectively.

Some beer was discovered recently walled up in the cellars of Messrs. Worthington, at Burton-on-Trent, which was brewed in 1798. At the last monthly house dinner of the Laboratory Club some of this beer was tasted and said to be sound. Its condition is described as being rather of the quality of sherry, and its colour as brilliant.

With a view to testing the accuracy of the statement that tuberculous meat is dangerous to health, some experiments were recently made at Munich. Eighteen guinea-pigs were injected with the juice of portions of the muscles of nine patients who died of phthisis, and though no tubercle was detected in the muscles used, fifteen of the guinea-pigs died of tuberculosis.

The *Journal de Medicine* gives a pitiful tale of ignorant cowardice. A farmer, who lately fell ill of smallpox, near Marseilles, was abandoned by his sister and children for fear of infection. The man became delirious, and went out on to the road, where he fell down. His courageous sons much too frightened to pick him up, contented themselves with covering the poor man with a straw mattress. Here he was left 36 hours exposed to sun, wind, and rain, and tormented by flies. At last a doctor heard of it, and with the aid of another man carried him into his cottage, where he died the next day.

Mr. Joseph Thomson, the African traveller, stated at Edinburgh a few days ago, that "he would unhesitatingly affirm in the plainest language that so far as our intercourse with the African race was concerned, instead of it being a blessing, it had been little better than an unmitigated curse to them. Taken as a whole, our trading stations on the greater part of the West Coast of Africa, instead of being centres of elevating influence, were centres of corruption, moral and physical."

These boisterous winds and the accompanying rain, of which we have had so much the last few days, are disagreeable even in the house, for the draught refuses to be kept out by the warmest of curtains and the most perfect of window fasteners. But those who are obliged to be out and about their business are the ones who enlist our sympathy. The minor delights of a wind storm, such as having our only hat hurled off into the gutter, to be made into a concertina by a passing hansom, or one's umbrella suddenly, without warning, turning itself inside out, these are mere nothings; it is the "cold" of every description which will catch us if it can in this sort of weather, which gives us a right to grumble. And to those who must be out exposed to the influence of wind and rain we give the same old advice—look to your boots, and remember the value of moderately-thick wool underclothing.

